# Marked PR interval variability in a patient with Brugada syndrome

**DOI:** 10.1097/j.pbj.0000000000000209

**Published:** 2023-04-10

**Authors:** Miguel Martins de Carvalho, Ricardo Alves Pinto, Tânia Proença, Delfim Souteiro, Luís Adão, Filipe Macedo, Manuel Campelo

**Affiliations:** a Department of Cardiology, São João Universitary Hospital, Oporto, Portugal; b Cardiovascular R&D Center, Universidade do Porto Faculdade de Medicina, Portugal.

To the Editor

The authors present a clinical case of a 54-year-old woman without relevant medical history other than Brugada type 1 pattern in the electrocardiogram, without chronic medication, who was referred for the cardiology outpatient clinic with palpitations. She had a strong family history of Brugada syndrome, but no history of sudden death, and she never had syncope. A transthoracic echocardiogram was performed, ruling out structural heart disease. Blood samples were analyzed, without thyroid function or electrolyte abnormalities. A 24-hour Holter examination was performed (digital Philips Zymed Holter, Model 1810 Plus Software, Philips Medical Systems) that revealed nocturnal periods of first-degree atrioventricular block (AVB) with a markedly variable PR interval and without nonconducted P waves; no other abnormalities were seen; the patient was asymptomatic. The P wave and QRS morphology were the same throughout the examination. The PR interval either increased or decreased, but it was always followed by a QRS complex. The number of P waves was the same as the QRS complexes. There was no relation between the PR interval variability and heart rate or circadian predominance. The patient is kept under follow-up in the outpatient clinic, uneventful. No genetic test was performed.

First-degree AVB is typically a benign situation^1^; however, it is associated with increased incidence of atrial fibrillation, heart failure, and mortality during follow-up.^2^ In some clinical settings, it can even be associated with markedly decreased survival.^3^

Some data point out that first-degree AVB on a basal ECG is an independent predictor of malignant arrhythmic events in Brugada syndrome (although this association was only demonstrated with first-degree AVB on basal ECG and not on Holter examination).^4,5^

The PR interval is highly affected by the autonomic nervous system.^6-9^ Sympathetic activation results in a decrease of the PR interval, whereas parasympathetic activation originates an increase of the PR interval.^6,7^ Previous studies showed that the increase in PR interval in response to vagal stimulation is well correlated with vagal stimulation frequency and can be regarded as linear.^7^

**Figure 1.**
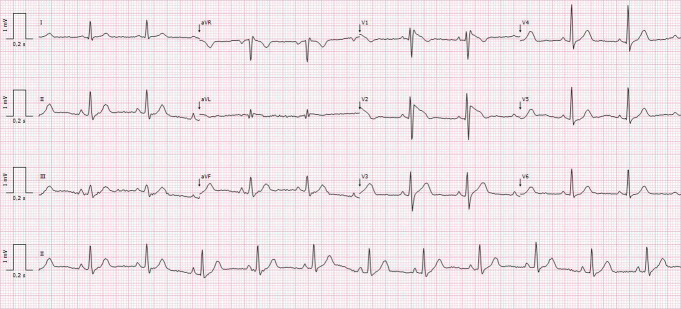
ECG revealing sinus rhythm with type 1 Brugada pattern.

**Figure 2.**
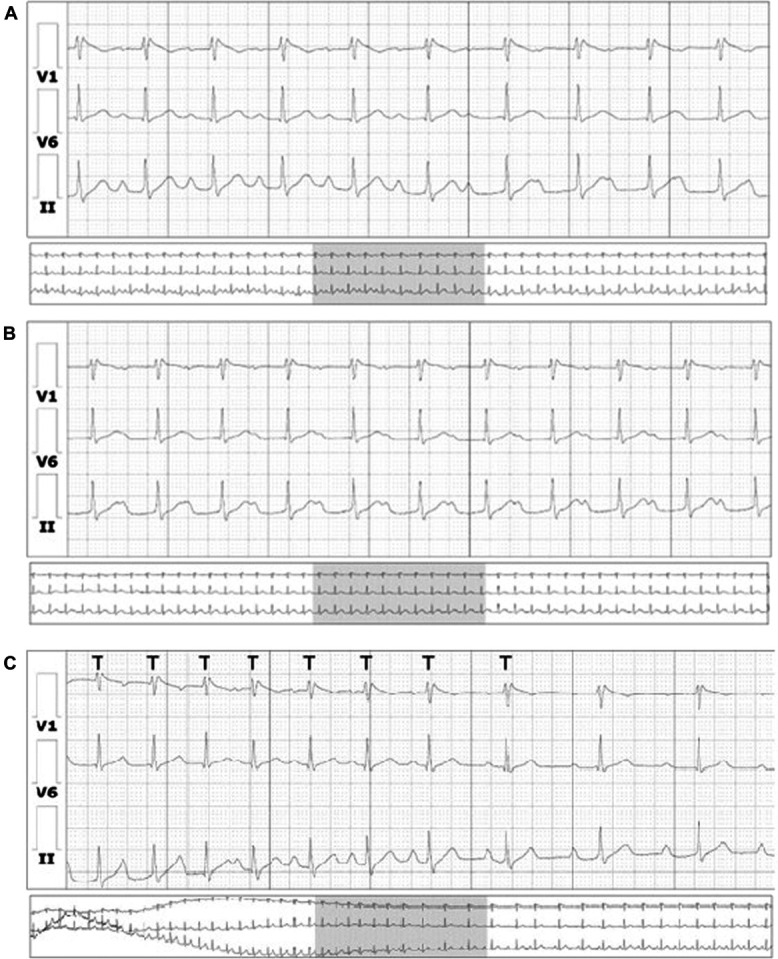
(A) Holter revealing sinus rhythm with type 2 Brugada pattern and first-degree AVB with a progressive PR interval prolongation; (B) continuation of previous Holter stripe; and (C) variable PR interval, always followed by a QRS complex. A type 1 Brugada pattern was not present in this examination.

The Brugada syndrome is diagnosed in the presence of a spontaneous type 1 pattern in patients without other heart disease, regardless of symptoms.^10^ It is a genetic sodium-related (I_Na_) channelopathy.^10^ The most common gene involved is the SCN5A, which is also associated with conduction diseases.^11^ Sodium currents are responsible for propagation of action potential through the myocardial cells. As patients with Brugada syndrome have reduced I_Na_ current, conduction disease is not unexpected to occur, if the SCN5A gene is involved.^12^ First-degree AVB could represent a marker of increased risk because of a stronger phenotype of I_Na_ reduction, leading to increased myocardial electrical instability.^5^ Other identified risk markers are the localization of type 1 Brugada pattern (Figs. 1 and 2), atrial fibrillation, fragmented QRS, QRS duration >120 ms, R wave in lead aVR, S wave in lead I (≥40 ms, amplitude ≥0.1 mV, area ≥1 mm^2^), early repolarization pattern in inferolateral leads, ST segment depression, T‐wave alternans, dispersion of repolarization, and Tzou criteria.^11^ In another recent study, the S wave in lead I was the only independent predictor of persistent risk of life-threatening arrhythmic events.^13^

The significance of this PR interval variability, with a “harmonic” appearance, is not yet known, as well as its clinical implications.
